# Beyond Hyperferritinemia: Evaluating Ferritin as a Predictor of Advanced Therapy in Adult-Onset Still’s Disease

**DOI:** 10.3390/diagnostics15202630

**Published:** 2025-10-18

**Authors:** Ji-Hyoun Kang

**Affiliations:** Division of Rheumatology, Department of Internal Medicine, Chonnam National University Hospital & Medical School, Gwangju 61469, Republic of Korea; romi918@naver.com

**Keywords:** adult-onset Still’s disease, ferritin, biomarker, prognosis, escalation

## Abstract

**Background:** Ferritin is a hallmark biomarker of systemic inflammation in adult-onset Still’s disease (AOSD), but its potential role in guiding escalation to advanced therapy has not been established. This study aimed to evaluate whether ferritin, alone or in combination with simple clinical variables, could predict the need for advanced therapy in AOSD. **Methods:** A retrospective review was conducted of 113 patients with AOSD fulfilling the Yamaguchi criteria at Chonnam National University Hospital. Baseline demographic, clinical, and laboratory data were collected at the index episode. The primary endpoint was defined as the use of advanced therapy—namely, intravenous immunoglobulin (IVIG), anakinra, or tocilizumab—during the index hospitalization. Ferritin was log-transformed and analyzed both as a continuous variable and in quartiles. A pragmatic Ferritin-Guided Escalation Rule (FGER) points score was constructed by combining ferritin quartiles with malignancy and ANA status. Logistic regression, Fisher’s exact test, and bootstrapped receiver operating characteristic (ROC) analyses were applied. **Results:** The cohort was predominantly male (64.6%) with a mean age of 44.9 years. Median ferritin was 4626.6 µg/L (IQR 1169.3–14,239.8). Advanced therapy was required in 15 patients (13.3%), including 14 who received tocilizumab, 1 IVIG, and 1 anakinra. When stratified by ferritin quartiles, advanced therapy occurred in 10.7% of Q1, 21.4% of Q2, 10.7% of Q3, and 10.7% of Q4 patients. Comparison of Q4 versus Q1–Q3 yielded an odds ratio of 0.67 (*p* = 0.47). Discriminatory performance was poor for both continuous ferritin (AUC 0.49, 95% CI 0.30–0.68) and the FGER points score (AUC 0.51, 95% CI 0.32–0.71). Calibration analysis also demonstrated limited agreement between predicted and observed risks. **Conclusions:** In this retrospective cohort, ferritin—whether assessed continuously, by quartiles, or within a simple bedside score—did not predict the need for advanced therapy (IVIG, anakinra, or tocilizumab) in AOSD. Although advanced therapies were administered to 13.3% of patients, ferritin was not a reliable escalation biomarker.

## 1. Introduction

Adult-onset Still’s disease (AOSD) is a rare, systemic autoinflammatory disorder that typically presents in young to middle-aged adults with quotidian fever, evanescent salmon-pink rash, arthritis or arthralgia, and lymphadenopathy, often accompanied by hepatosplenomegaly and other systemic features [1,2]. The clinical spectrum is heterogeneous, encompassing monophasic, polycyclic, and chronic articular or systemic patterns, which complicates disease management and long-term prognosis [3]. Severe, life-threatening complications—most notably macrophage activation syndrome (MAS), a form of secondary hemophagocytic lymphohistiocytosis (HLH) associated with AOSD—as well as disseminated intravascular coagulation, remain key determinants of morbidity and mortality [4].

Among laboratory abnormalities, serum ferritin is considered the hallmark biomarker of AOSD. Hyperferritinaemia, frequently reaching values several-fold higher than in other inflammatory conditions, is so characteristic that ferritin has been incorporated into both Yamaguchi and Fautrel classification criteria as well as several disease activity scores [5,6,7]. Ferritin levels exceeding 3000–5000 ng/mL are often reported, and extreme hyperferritinaemia (>10,000 ng/mL) is highly suggestive of AOSD in the appropriate clinical context [8]. Beyond diagnostic value, high ferritin has been linked to systemic complications, including MAS, and to a more aggressive disease phenotype, raising interest in its potential role as a prognostic marker [9,10]. However, whether ferritin can serve as a biomarker to guide therapeutic decision-making, particularly escalation to advanced therapy, remains unresolved.

The therapeutic landscape of AOSD has evolved considerably. Initial management typically relies on non-steroidal anti-inflammatory drugs (NSAIDs) and systemic glucocorticoids, which remain the cornerstone of acute disease control [11]. Conventional synthetic disease-modifying anti-rheumatic drugs (csDMARDs), such as methotrexate or azathioprine, are commonly used as steroid-sparing agents in refractory or chronic disease [12]. For patients with inadequate response or severe systemic involvement, escalation to advanced therapies—including intravenous immunoglobulin (IVIG) and biologic agents targeting interleukin (IL)-1 or IL-6 pathways (anakinra, tocilizumab)—is increasingly adopted [13,14,15]. Biologics have transformed outcomes for a subset of patients, yet the decision of when to escalate therapy is often based on clinical severity or physician judgment rather than validated biomarkers [16].

Despite routine ferritin measurement in nearly all suspected or confirmed cases, its predictive role in guiding treatment escalation has not been formally validated. A widely accessible biomarker such as ferritin could be of substantial clinical relevance if it enabled early identification of patients who require aggressive intervention, thereby reducing morbidity and preventing life-threatening complications. Prior studies have primarily focused on ferritin as a diagnostic marker or indicator of disease activity, while relatively few have systematically assessed its role in predicting escalation to IVIG or biologics [17].

Unlike prior reports that primarily focused on ferritin as a diagnostic or disease-activity marker, this study specifically examined its role as a pragmatic predictor for treatment escalation in real-world AOSD care. By quantifying its predictive limits and proposing a simple composite framework, the study contributes practical insights for designing future escalation algorithms.

Therefore, this retrospective study aimed to investigate whether serum ferritin, alone or integrated into a pragmatic point-based score with clinical parameters, could predict the need for advanced therapy in patients with AOSD. By clarifying the utility of ferritin as a biomarker beyond diagnosis, this study sought to provide insight into a more evidence-based approach to therapeutic decision-making in AOSD.

## 2. Methods

This study was designed as a retrospective, observational cohort study including consecutive adult patients with AOSD who received care at Chonnam National University Hospital (CNUH), a tertiary referral center in South Korea. Eligible patients were identified through hospital records spanning the study period, and only those with sufficient clinical, laboratory, and treatment data available in their electronic medical records were included in the analysis. The study protocol was reviewed and approved by the Institutional Review Board of CNUH on 25 February 2020 (approval number: CNUH-2020-043). Given the retrospective nature of the study and the exclusive use of anonymized data extracted from existing records, the requirement for written informed consent from patients was formally waived.

All procedures were carried out in compliance with the ethical principles of the Declaration of Helsinki and relevant national regulations regarding research with human participants. The conduct and reporting of the study adhered to the Strengthening the Reporting of Observational Studies in Epidemiology (STROBE) guidelines, ensuring methodological transparency and reproducibility.

### 2.1. Study Population and Eligibility Criteria

Adults aged 18 years or older who ultimately satisfied the internationally recognized Yamaguchi classification criteria for AOSD [2] and who had sufficient clinical and laboratory data available in the electronic medical records were considered eligible for inclusion. To enhance diagnostic specificity, the study carefully excluded individuals who were subsequently found to have alternative explanations for their presentation, including infectious diseases, malignant conditions, or other systemic rheumatic disorders that can mimic AOSD in their early course. Patients who lacked essential baseline demographic, clinical, or laboratory variables were also excluded in order to ensure robustness of subsequent analyses.

For the purposes of the present biomarker investigation, the index episode was defined operationally as the point at which the Yamaguchi criteria were first fulfilled or, alternatively, the index hospital admission attributed to AOSD in cases where diagnosis was established in an inpatient setting. This definition provided a standardized temporal anchor for biomarker measurement and outcome ascertainment. Patients without a recorded ferritin measurement at or near the index episode were excluded from ferritin-based analyses, as the absence of this key biomarker would preclude meaningful evaluation. Together, these eligibility criteria were designed to create a cohort with diagnostic rigor, adequate data completeness, and a consistent reference time point for subsequent analyses.

### 2.2. Data Collection

Demographic variables collected for each patient included age at disease onset, sex, and disease duration from the time of symptom onset to the index episode, thereby allowing characterization of both acute and chronic patterns of presentation. Clinical manifestations were systematically reviewed at baseline or during the index episode and encompassed a broad spectrum of systemic features. These included high spiking quotidian fever and the characteristic evanescent, salmon-colored skin rash, together with musculoskeletal features such as arthritis or arthralgia. Additional systemic signs were documented, including lymphadenopathy, hepatocellular dysfunction or cholestasis reflecting liver involvement, and constitutional symptoms such as sore throat and myalgia. Evidence of organomegaly (splenomegaly and/or hepatomegaly) was recorded, along with serosal involvement (pericarditis, pleuritis) and severe complications including acute respiratory distress syndrome (ARDS), hemophagocytic lympho-histiocytosis (HLH), and MAS.

Laboratory variables were extracted from electronic records at the time of the index episode. These included a complete blood count (CBC) with differential, with specific attention to the absolute neutrophil count, as well as liver function tests assessing both hepatocellular and cholestatic enzymes. Serum ferritin levels were central to the analysis, and inflammatory markers such as erythrocyte sedimentation rate (ESR) and C-reactive protein (CRP) were also measured. Autoantibody data were collected where available, including rheumatoid factor (RF), anti-cyclic citrullinated peptide (anti-CCP), and antinuclear antibody (ANA), to help exclude mimicking rheumatologic conditions and to provide additional immunologic context. Serum ferritin levels were available to investigators during hospitalization. For analytic consistency, the maximum ferritin value recorded during the index hospitalization was selected as the representative biomarker for each patient, as this measure most accurately reflects the peak inflammatory burden and aligns with previous AOSD literature.

Therapeutic data were comprehensively reviewed. Use of non-steroidal anti-inflammatory drugs (NSAIDs) at diagnosis was recorded, as well as glucocorticoid therapy characterized by daily mean, cumulative, and maximal prednisolone-equivalent doses. Exposure to conventional synthetic disease-modifying anti-rheumatic drugs (csDMARDs), including methotrexate, azathioprine, and calcineurin inhibitors, was documented. Advanced therapies were also captured, encompassing intravenous immunoglobulin (IVIG) and biologic agents. The latter included tumor necrosis factor (TNF) inhibitors, as well as cytokine-targeted agents such as anakinra (IL-1 receptor antagonist) and tocilizumab (IL-6 receptor antagonist). These therapeutic variables allowed assessment of escalation patterns and evaluation of predictors of advanced treatment use.

### 2.3. Outcome Definition

The primary outcome of interest in this study was the need for escalation to advanced therapy. This endpoint was defined a priori to ensure objectivity and consistency across the cohort. Specifically, advanced therapy was operationalized as the initiation of either IVIG, the IL-1 receptor antagonist anakinra, or the IL-6 receptor inhibitor tocilizumab, administered at any point during the patient’s index hospitalization or index disease episode. By defining the outcome at the outset, the study avoided post hoc reclassification and maintained alignment with clinically meaningful interventions.

This outcome was chosen deliberately to capture treatment escalation beyond conventional management. In routine practice, NSAIDs, glucocorticoids, and csDMARDs often represent the first tiers of therapy, while IVIG and cytokine-targeted biologics are reserved for patients with severe systemic manifestations, refractory disease, or intolerance to standard agents. As such, the use of IVIG, anakinra, or tocilizumab provides a pragmatic surrogate marker for clinically significant therapeutic escalation, reflecting both physician judgment and patient need. Restricting the outcome window to the index episode ensured that exposure reflected acute decision-making during the initial disease course, rather than later treatment shifts influenced by chronicity, cumulative toxicity, or evolving practice patterns.

Framing the endpoint in this way enabled the study to evaluate whether baseline clinical or laboratory variables—including ferritin—had predictive value for early escalation to therapies typically reserved for the most challenging disease courses. This clear and clinically relevant definition strengthened the interpretability of the findings and underscored the translational importance of the biomarker analyses.

### 2.4. Statistical Analysis

Baseline characteristics were summarized using means with standard deviations (SD) or medians with interquartile ranges (IQR) for continuous variables, and counts with percentages for categorical variables. Serum ferritin was markedly right-skewed and therefore transformed as log(1+ferritin) for analyses. Patients were divided into equal-count quartiles of log-ferritin, and advanced-therapy rates were compared across quartiles. To evaluate whether the highest ferritin quartile (Q4) was associated with escalation compared with the lower quartiles (Q1–Q3), this study applied Fisher’s exact test with a one-sided alternative hypothesis (greater odds in Q4). The corresponding untransformed ferritin ranges were approximately Q1 (<1200 µg/L), Q2 (1200–4000 µg/L), Q3 (4000–10,000 µg/L), and Q4 (≥10,000 µg/L). Each quartile contained 28 patients (*n* = 112 in total).

The logistic regression model included three predictors (log-ferritin, ANA, and malignancy), yielding an event-per-variable ratio of approximately 5, which is within the acceptable lower bound for exploratory analyses. Given the limited event count (*n* = 15), AUC estimates should be interpreted with caution but remain informative for hypothesis generation.

**Figure 1:**
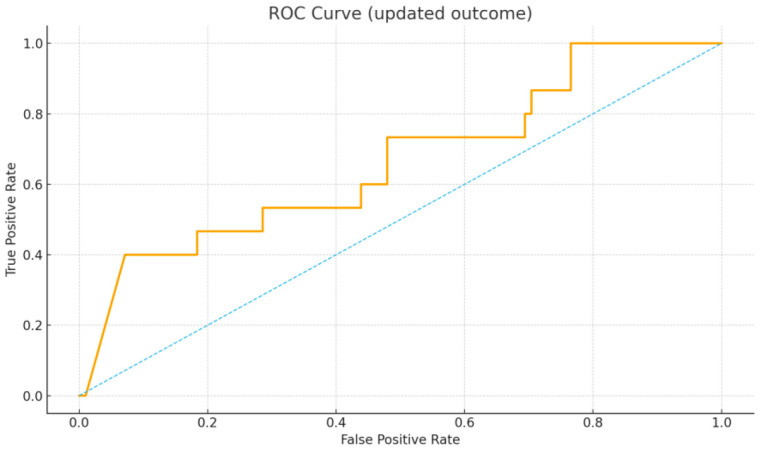
Receiver operating characteristic (ROC) curve from logistic regression including log-ferritin, ANA, and malignancy (outcome defined as IVIG/anakinra/tocilizumab).

**Figure 2:**
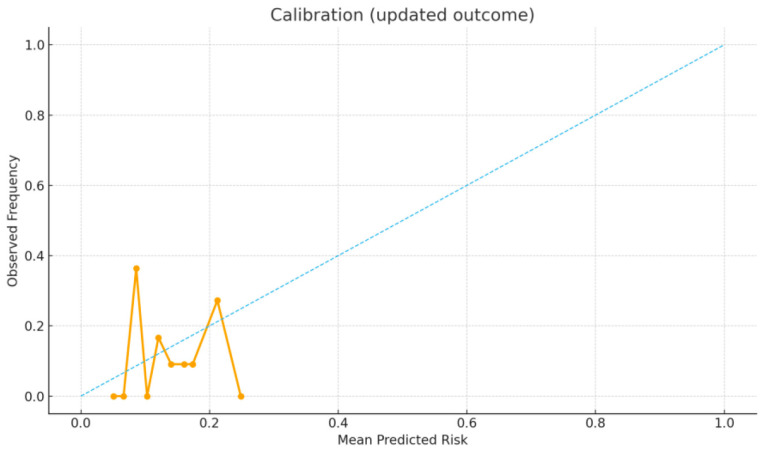
Calibration plot (10 quantile bins) showing observed versus predicted probabilities of advanced therapy; the diagonal indicates perfect calibration.

The present study also constructed a pragmatic Ferritin-Guided Escalation Rule (FGER) points score, assigning 0–3 points by ferritin quartile (Q1 = 0, Q2 = +1, Q3 = +2, and Q4 = +3), with additional points for malignancy (+1) and ANA negativity (+1). Discrimination of continuous ferritin and of the FGER score was assessed using the area under the receiver operating characteristic curve (AUC) with 95% confidence intervals (CIs) estimated by percentile bootstrap resampling (B = 500), retaining only resamples with both outcome classes. To illustrate model performance, this study additionally fit an exploratory logistic regression model including log-ferritin, ANA, and malignancy, and generated predicted probabilities to plot the ROC curve (Figure 1) and a calibration curve using 10 quantile bins (Figure 2). The calibration curve displayed observed frequencies against mean predicted probabilities, with the 45° diagonal line representing perfect calibration.

Given the limited number of outcome events, all analyses were considered exploratory and interpreted cautiously. All statistical analyses were performed using Python software version 3.11.5 (*Python Software Foundation, Wilmington, DE, USA*), employing the pandas (version 2.2.2) and numpy (version 1.26.4) libraries for data management, statsmodels (version 0.14.2) for logistic regression, scikit-learn (version 1.4.0) for ROC/AUC estimation, bootstrap resampling, and calibration curves, and matplotlib (version 3.8.4) for figure generation. A fixed random seed was applied to ensure reproducibility, with the choice of value being arbitrary and carrying no analytic meaning.

## 3. Results

A total of 113 patients with adult-onset Still’s disease (AOSD) were included. The mean age was 44.9 ± 14.9 years, and the median age was 44.0 years (interquartile range [IQR] 33.8–55.5). Of these, 73 (64.6%) were male and 40 (35.4%) were female. Serum ferritin was available for 112 patients, with a median of 4626.6 µg/L (IQR 1169.3–14,239.8; range 24.5–77,073). Antinuclear antibody (ANA) was tested in 112 patients, all of whom were negative. A history of malignancy was present in two patients (1.8%). With respect to treatment, IVIG was administered in 1 patient (0.9%), anakinra in 1 patient (0.9%), and tocilizumab in 13 patients (11.5%). In total, 15 patients (13.3%) received advanced therapy (Table 1). When IVIG and cytokine blockers were analyzed separately, both subgroups showed similarly weak associations between ferritin level and escalation, consistent with the main analysis; however, small event counts precluded formal statistical testing. As shown in Supplement Table S1, baseline demographic, clinical, and laboratory characteristics were similar between the advanced-therapy and non-advanced groups. None of the evaluated parameters, including ferritin and inflammatory markers, differed significantly between the two groups.

When stratified into equal-count quartiles of log-transformed ferritin, advanced therapy occurred in 3 of 28 patients in Q1 (10.7%), 6 of 28 in Q2 (21.4%), 3 of 28 in Q3 (10.7%), and 3 of 28 in Q4 (10.7%) (Table 2). Thus, events were distributed across quartiles, but no consistent gradient was observed with increasing ferritin.

A direct comparison of the highest quartile (Q4) versus the lower quartiles (Q1–Q3 combined) is presented in Table 3. Advanced therapy occurred in 3 of 29 patients in Q4 compared with 12 of 83 in Q1–Q3, yielding an odds ratio of 0.67 with a one-sided *p*-value of 0.47. This indicates that higher ferritin was not associated with an increased likelihood of escalation.

The discriminatory performance of ferritin as a continuous variable and of the Ferritin-Guided Escalation Rule (FGER) points score is shown in Table 4. Continuous ferritin yielded an AUC of 0.49 (95% CI 0.30–0.68), while the FGER points score yielded an AUC of 0.51 (95% CI 0.32–0.71). Both metrics demonstrated performance close to chance with wide confidence intervals. The ROC curve of the multivariable logistic regression model, including log-ferritin, ANA, and malignancy, is depicted in Figure 1, illustrating limited discrimination.

The distribution of FGER points and corresponding event rates is presented in Table 5. Among patients with 0 points (*n* = 7), none required advanced therapy. For those with 1 point (*n* = 30), 5 (16.7%) received advanced therapy; for 2 points (*n* = 22), 4 (18.2%); for 3 points (*n* = 30), 5 (16.7%); and for 4 points (*n* = 24), 1 (4.2%). No monotonic increase in advanced-therapy rates was observed across point strata. The calibration plot (Figure 2) demonstrated poor alignment between predicted probabilities and observed frequencies, consistent with the limited predictive value of the model.

Model discrimination was evaluated using a multivariable logistic regression including log-transformed ferritin, ANA, and malignancy. The receiver operating characteristic (ROC) analysis showed limited discrimination (AUC ≈ 0.5), indicating no meaningful separation between patients who did and did not require advanced therapy. Calibration across 10 quantile bins demonstrated wide deviation from the ideal diagonal, confirming poor agreement between predicted and observed probabilities. Together, these findings indicate that ferritin-based models lack sufficient predictive accuracy for guiding escalation decisions.

## 4. Discussion

In this retrospective cohort of patients with AOSD, extreme hyperferritinaemia was observed frequently, confirming its status as a characteristic laboratory hallmark of the condition. However, serum ferritin—whether analyzed as a continuous variable, categorized into quartiles, or incorporated into a pragmatic point-based Ferritin-Guided Escalation Rule (FGER)—did not demonstrate meaningful ability to predict subsequent escalation to advanced therapy, defined as the initiation of intravenous immunoglobulin, anakinra, or tocilizumab. Even when the therapeutic endpoint was broadened to explicitly include tocilizumab, escalation was recorded in 13.3% of the cohort, and these events were distributed relatively evenly across ferritin quartiles. Notably, patients in the highest quartile did not exhibit a higher likelihood of requiring advanced therapy compared with those in the lower three quartiles combined. Overall, ferritin—despite its established diagnostic and activity-related role—did not perform as a predictive biomarker for therapeutic escalation.

Several explanations are plausible for the observed lack of predictive value of ferritin. First, the clinical decision to initiate advanced therapy in AOSD is inherently multifactorial and extends well beyond a single laboratory parameter. Escalation may be driven by features that are only partially correlated with systemic ferritin levels—such as refractory arthritis, persistent serosal involvement, hepatic dysfunction, pulmonary manifestations, or clinician judgment regarding the urgency of steroid-sparing strategies—thereby introducing confounding by indication into observational datasets. Second, ferritin likely behaves primarily as a state marker of systemic inflammation rather than as a reliable predictive marker of treatment responsiveness. While prior studies have consistently demonstrated associations between elevated ferritin, systemic disease activity, and MAS, translating these associations into a tool for therapeutic triage has remained elusive. Third, heterogeneity of disease phenotype may obscure linear relationships between ferritin and escalation; for instance, tocilizumab is often used in articular or glucocorticoid-dependent forms with only moderate ferritin elevation, while patients with extreme levels may respond to corticosteroids alone. Finally, reliance on a single time point ferritin measurement may miss informative longitudinal dynamics. Biomarker trajectories—including the absolute change (delta ferritin), the rate of rise or decline, or failure to normalize over time—may offer greater prognostic insight into impending deterioration than any isolated value. Future research incorporating serial ferritin measurements, in combination with other inflammatory biomarkers and clinical features, could therefore be more informative for predicting escalation to biologic or adjunctive therapies.

The absence of predictive value for ferritin alone should not be misconstrued as evidence that the marker lacks clinical utility. Ferritin remains indispensable for classification, differential diagnosis, and disease activity monitoring, and very high levels should continue to trigger urgent evaluation for MAS and other hyperinflammatory states. Nevertheless, escalation decisions should not rely solely on ferritin but integrate it into a broader clinical context.

Composite approaches warrant prioritization. First, established clinical systemic scores (e.g., the Pouchot systemic score) incorporate fever, rash, lymphadenopathy, hepatosplenomegaly, and serositis, thereby integrating the clinical breadth of disease expression [12]. Second, laboratory composites deserve exploration. In addition to ferritin, parameters such as platelet count, liver transaminases, CRP, ESR, lactate dehydrogenase (LDH), fibrinogen, triglycerides, and soluble interleukin-2 receptor (sIL-2R) have all been implicated in hyperinflammation and may provide additive prognostic value [8,18]. The glycosylated ferritin fraction (<20%), a feature more specific to AOSD than total ferritin, has also demonstrated diagnostic and prognostic relevance [19,20]. Third, multiparametric risk tools developed for hyperinflammatory syndromes, such as the HS score for secondary haemophagocytic lymphohistiocytosis (HLH), may have translational value in identifying patients with AOSD at risk of severe systemic complications and in guiding timely escalation [21].

Beyond these, cytokine-related biomarkers represent promising adjuncts. Interleukin-18 (IL-18), for instance, has shown strong associations with systemic activity and MAS development, while calprotectin (S100A8/A9) and S100A12 have been repeatedly linked to systemic flares and poor outcomes [20,21,22]. Future studies should evaluate composite and dynamic markers prospectively to enhance early recognition and personalize escalation strategies.

The strengths of this study merit highlighting. The analysis was based on a clearly defined cohort of patients who all satisfied validated classification criteria for AOSD, ensuring diagnostic homogeneity and minimizing misclassification. Uniform outcome ascertainment within the index episode reduced the risk of biased case capture, and the primary analyses were re-conducted using a clinically meaningful endpoint that incorporated tocilizumab, thereby aligning the outcome more closely with current therapeutic practice. Together, these design features support the internal validity of the findings.

Nevertheless, important limitations require emphasis. The retrospective, single-center design inevitably restricts generalizability to broader populations and leaves the study vulnerable to unmeasured confounding, including variability in therapeutic availability, institutional protocols, and individual clinician preferences [11]. Although broadening the endpoint definition increased the number of observed events, the absolute event count remained modest. This limitation constrains statistical power, contributes to wide confidence intervals, and underscores that null or near-chance discrimination estimates should be interpreted with caution rather than definitive absence of effect. Defining escalation as the use of advanced therapy may introduce operator bias, as treatment decisions are influenced by physician judgment, institutional practice, and drug accessibility. The dataset did not include standardized definitions of treatment failure or major organ damage, which would have strengthened outcome validity. Additionally, while our primary analysis focused on the positive predictive value of ferritin, low ferritin values also failed to reliably identify patients who would not require escalation, highlighting the absence of both positive and negative predictive discrimination.

In addition, ferritin was assessed as a single baseline measurement without standardized sampling windows. The absence of kinetic data (e.g., rates of rise or fall, or persistence of hyperferritinaemia) may have attenuated true associations that could only be captured through serial monitoring. Several biologically relevant candidate predictors—including fibrinogen, triglycerides, glycosylated ferritin, IL-18, and sIL-2R—were either unavailable or incomplete in the dataset, precluding the development of richer multivariable models that might more accurately predict escalation [12,18,19,20]. Finally, escalation as defined by receipt of advanced therapy is at best an imperfect proxy for clinical need. Treatment initiation reflects not only disease severity but also local practice patterns, physician judgment, patient preference, and drug accessibility, which may differ across centers and healthcare systems [13,14]. These contextual influences, although difficult to quantify, must temper interpretation and emphasize the need for replication in larger, multicenter, and ideally prospective cohorts.

Clinically, these findings underscore that ferritin alone should not be used as a sole trigger for initiating advanced therapy in AOSD. Instead, a more defensible approach is to integrate ferritin levels with the broader clinical phenotype and complementary biomarkers, applying escalation criteria that are pre-specified and clinically meaningful—such as steroid-refractory quotidian fever, persistent organ dysfunction, or early warning features of MAS. Embedding such criteria within a treat-to-target framework may help harmonize decision-making across centers, reduce variation in practice, and optimize timing of escalation [23]. Importantly, this framework aligns with broader rheumatology strategies, in which composite indices are routinely used to balance laboratory findings with clinical presentation.

From a research perspective, the results highlight the need for larger, multicenter cohorts with standardized sampling protocols and prospective capture of biomarker kinetics, treatment decision thresholds, and patient outcomes. Pragmatic designs—such as registry-based longitudinal studies, multicenter collaborations, or adaptive observational cohorts—are particularly well suited to quantify how composite biomarker strategies might influence the timing of escalation, cumulative glucocorticoid exposure, MAS incidence, and patient-centered outcomes such as functional status or quality of life [24]. Integration of real-world data with harmonized definitions could further enable cross-cohort comparisons and accelerate biomarker validation.

Finally, the value of negative findings should be acknowledged. Demonstrating that ferritin does not reliably predict escalation clarifies its appropriate role in clinical care and sharpens the focus of future biomarker research. Negative results reduce futile testing of inadequately informative markers, mitigate publication bias, and help recalibrate expectations about what ferritin can—and cannot—achieve in the management of AOSD [25]. By delineating the boundaries of ferritin’s utility, this study contributes constructively to the incremental refinement of evidence-based, precision-oriented care in systemic autoinflammatory disease.

## 5. Conclusions

In conclusion, ferritin remains a readily accessible gauge of systemic inflammatory burden in AOSD; however, considered in isolation, it does not discriminate which patients will require advanced therapy—intravenous immunoglobulin, anakinra, or tocilizumab—during the index episode. Future research should prioritize integrated models that combine ferritin kinetics with cytokine markers such as IL-18 and S100A8/A9, systemic inflammation indices, and clinical trajectories within a precision medicine framework. Such multidimensional tools may improve timing of escalation, reduce glucocorticoid exposure, and align with treat-to-target principles increasingly applied in rheumatology.

## Figures and Tables

**Table 1:** Baseline characteristics of total patients.

Characteristic	Value
*N*	113
Age, mean (SD)	44.9 (14.9)
Age, median (IQR)	44.0 (33.8–55.5)
Male, *n* (%)	73 (64.6%)
Female, *n* (%)	40 (35.4%)
ANA positive, *n*/*N* (%)	22/112 (19.6%)
Malignancy, *n*/*N* (%)	2/113 (1.8%)
Ferritin, median (IQR)	4626.6 (1169.2–14,239.8)
Ferritin, min–max	24.54–77,073.00
IVIG, *n* (%)	1 (0.9%)
Anakinra, *n* (%)	1 (0.9%)
Tocilizumab, *n* (%)	13 (11.5%)
Advanced therapy (IVIG/anakinra/tocilizumab), *n* (%)	15 (13.3%)

**Table 2:** Advanced-therapy use across quartiles of log-transformed ferritin.

Quartile	*N*	Advanced *n*	Advanced Rate
Q1	28	4	0.1428571428571428
Q2	28	2	0.0714285714285714
Q3	28	2	0.0714285714285714
Q4	28	7	0.25

**Table 3:** Comparison of the highest ferritin quartile (Q4) versus lower quartiles (Q1–Q3) for advanced therapy (Fisher’s exact test, one-sided).

Comparison	Advanced *n*/*N*	Odds Ratio (Fisher Exact)	*p*-Value (One-Sided)
Q4 vs. Q1–Q3	7/28 vs. 8/84	3.1666666666666665	0.0439276760663717

**Table 4:** Discriminatory performance (AUC with bootstrap 95% CI) of ferritin (continuous) and FGER points score.

Model	AUC	AUC 95% CI Low	AUC 95% CI High
Ferritin (continuous)	0.6472767790925871	0.4506141161966405	0.8280172768408062
FGER points (ordinal)	0.6110964313427918	0.445060292240034	0.7731972639011472

**Table 5:** Distribution of FGER points and corresponding advanced-therapy rates.

FGERPoints	*N*	Advanced *n*	Advanced Rate
0.0	7.0	0.0	0.0
1.0	30.0	4.0	0.1333333333333333
2.0	22.0	2.0	0.0909090909090909
3.0	30.0	3.0	0.1
4.0	24.0	6.0	0.25

## Data Availability

The data underlying this article are not publicly available due to institutional restrictions and patient confidentiality. De-identified datasets may be made available from the corresponding author on reasonable request and with permission of the Institutional Review Board.

## Supplementary Materials

The following supporting information can be downloaded at: https://www.mdpi.com/article/10.3390/diagnostics15202630/s1, Table S1. Baseline characteristics according to advanced-therapy status.
