# Supplementary material for: Beyond Hyperferritinemia: Evaluating Ferritin as a Predictor of Advanced Therapy in Adult-Onset Still’s Disease
Source: Diagnostics (Basel). 2025 Oct 18;15(20):2630. doi: 10.3390/diagnostics15202630 (PMC12564851; doi:10.3390/diagnostics15202630)
Supplement: Supplementary file 1 [file diagnostics-15-02630-s001.zip › diagnostics-3894931-supplementary.pdf]

Supplement Table S1. Baseline characteristics according to advanced-therapy status

Variable	Advanced therapy (n=15)	Non-advanced (n=98)	p-value
Age, years, mean ± SD	45.2 ± 13.8	44.8 ± 15.0	0.92
Male sex, n (%)	10 (66.7)	63 (64.3)	0.85
Fever, n (%)	15 (100)	95 (96.9)	0.52
Rash, n (%)	11 (73.3)	74 (75.5)	0.86
Arthritis/arthralgia, n (%)	9 (60.0)	60 (61.2)	0.93
Lymphadenopathy, n (%)	3 (20.0)	19 (19.4)	0.97
Hepatosplenomegaly, n (%)	5 (33.3)	31 (31.6)	0.89
Serositis (pleuritis/pericarditis), n (%)	2 (13.3)	12 (12.2)	0.91
MAS, n (%)	2 (13.3)	10 (10.2)	0.73
Ferritin, µg/L, median (IQR)	5890 (2200–15100)	4450 (1100–13900)	0.47
ESR, mm/h, median (IQR)	58 (37–93)	61 (40–96)	0.76
CRP, mg/L, median (IQR)	82.5 (46.8–145.0)	79.0 (41.0–141.0)	0.84
AST, U/L, median (IQR)	67 (35–126)	59 (32–121)	0.67
ALT, U/L, median (IQR)	58 (31–115)	53 (29–104)	0.71
Platelet count, $\times 10^9$/L, mean ± SD	238 ± 110	245 ± 107	0.81
WBC, $\times 10^9$/L, mean ± SD	12.3 ± 5.1	11.8 ± 4.9	0.72
Neutrophil count, $\times 10^9$/L, mean ± SD	10.5 ± 4.8	9.9 ± 4.7	0.64
ANA positivity, n (%)	0 (0)	0 (0)	–
